# Age and sex associations of SARS-CoV-2 antibody responses post BNT162b2 vaccination in healthcare workers: A mixed effects model across two vaccination periods

**DOI:** 10.1371/journal.pone.0266958

**Published:** 2022-04-29

**Authors:** Cleo Anastassopoulou, Dimitra Antoni, Yiannis Manoussopoulos, Panagiotis Stefanou, Sofia Argyropoulou, Georgia Vrioni, Athanasios Tsakris

**Affiliations:** 1 Department of Microbiology, Medical School, National and Kapodistrian University of Athens, Athens, Greece; 2 Department of Microbiology, General Hospital of Arta, Arta, Greece; 3 Laboratory of Virology, ELGO-Demeter, Patras, Greece; IAVI, UNITED STATES

## Abstract

This study aimed to examine the associations with epidemiological, behavioral and clinical parameters of IgG antibody responses against the spike protein of severe acute respiratory syndrome coronavirus 2 (SARS-CoV-2) after immunization with two doses of the BNT162b2 vaccine in a cohort of healthcare workers (HCWs, n = 439) in Greece. We used a mixed effects model to investigate the potential associations of antibody levels one and three months after vaccination and examined by bootstrapping t-tests the putative effects of gender and age for each period. We also employed exact tests of independence in R × C contingency tables to explore associations between behavioral and gender variables with vaccinations side effects. We found significant differences between males and females as well as between subjects in the youngest (21–30 years) and the older age groups in both study periods. We also detected a decrease in titers with age and time. Males had steeper elimination rates across the age span in both periods, in contrast to females who exhibited a softer elimination titer rate with age in the first period and almost constant titers in the second. Concerning side effects, we found a significant association between pain at the injection site and female sex. Hence, our real-world data analyses revealed potentially important clues into the associations of antibody responses to SARS-CoV-2 spike. We discuss the importance of these findings in view of current mass vaccination perspectives and provide useful clues for the design and optimal timing of booster doses for COVID-19.

## Introduction

The swift development of several highly effective vaccines that mitigate the devastating consequences of the coronavirus disease 2019 (COVID-19) pandemic just over a year after the emergence of severe acute respiratory syndrome coronavirus 2 (SARS-CoV-2), restored humanity’s optimism for an accelerated return to a new normalcy. Pfizer-BioNTech’s BNT162b2 (Comirnaty, Tozinameran), which is based on the novel, readily adaptable platform of messenger RNA (mRNA), was the first vaccine that was granted emergency use authorization in late December 2020, full approval recently in individuals 16 years of age and older and emergency use authorization in children aged 12–15 [1]. BNT162b2 proved to be safe and highly (95%) efficacious in clinical trials and in real-world settings, as indicated by the 94% and 92% effectiveness against symptomatic infection and severe COVID-19, respectively, identified in Israel that was leading the vaccination race (with the highest proportional rate of COVID-19 vaccine administration for its population) until recently [2–4].

The first large-scale study to evaluate the antibody response to the BNT162b2 vaccine in HCWs in Israel across different ages, sexes, and comorbidities found that almost all study participants developed IgG and neutralizing antibodies that were highly correlated, rapidly after two vaccine doses [5]. Several questions nonetheless remain regarding the correlates of protection of the novel COVID-19 vaccines, the kinetics of anti-SARS-CoV-2 antibodies after vaccine administration and the factors that may influence them [6,7]. Available data include preliminary kinetics limited to a three case-series [8] and a more comprehensive sequential serum sample cohort of 180 Finnish healthcare workers (HCWs) tested at weeks 3 and 6 post vaccination that included IgG, IgA, and IgM antibody responses so as to assess neutralization activity against different SARS-CoV-2 variants [9].

Grupel *et al*., who studied SARS-CoV-2 anti-spike IgG kinetics in 116 Israeli HCWs receiving BNT162b2, found a statistically significant difference in IgG levels between subjects younger than 50 years and older individuals [10]. Müller *et al*. compared antibody responses between two age groups of vaccinees, younger than 60 and older than 80 years and found lower frequencies of neutralizing antibodies in the elderly group [11]. Neither of these studies considered the effects of biological sex, although sex-based immunological differences may contribute to variations in responses to vaccines in males and females [12]. Michos *et al*. did not find statistically significant differences in immune responses between the sexes regarding either total or neutralizing antibodies against the receptor binding domain (RBD) of SARS-CoV-2 spike protein for both vaccines’ doses in a cohort of 268 HCWs; in contrast, older age, smoking, and immunosuppressive medications were reported to negatively affect antibody levels after immunization with the BNT162b2 vaccine [13].

In the literature, there is agreement on the waning of adaptive humoral immune responses with time from vaccination against COVID-19. For instance, Favresse *et al*. observed a significant antibody decline three months post-vaccination with BNT162b2 in their study of 142 sero-negative individuals, without reporting the effects of gender and age on the response [14]. The decline of antibody titers three months after two doses of BNT162b2 in non-immunocompromised adults was also confirmed by other studies [e.g. [15]].

The analysis of emerging serological data following vaccination enhances our understanding of the immunological response to the novel COVID-19 vaccines and the factors that may influence them, thereby informing public health policy decisions [13]. This study aimed to assess the associations of antibody responses against the RBD of the spike protein of SARS-CoV-2 after immunization with two doses of the BNT162b2 vaccine and three months post-vaccination, with epidemiological, behavioral and clinical parameters in a cohort of HCWs.

## Materials and methods

### Study participants and design

Sera were collected between February and May, 2021 on a voluntary basis from 458 HCWs of the General Hospital of Arta, a public hospital in northwestern Greece providing primary and secondary health services. Of those, twelve had been previously infected with SARS-CoV-2 as determined by a confirmed positive RT-PCR result and were thus excluded from vaccination (and the study), whereas seven subjects who had not been previously infected or vaccinated served as negative controls in the serological assay. The remaining 439 individuals who received two doses of Pfizer-BioNTech’s BNT162b2 (Comirnaty, Tozinameran) 21 days apart according to the recommended schedule, were enrolled in this study. A form containing demographic and clinical data as well as adverse events (AEs) after each vaccine dose was completed by each participant. Data collected at baseline included gender, age, and basic medical history that entailed information on underlying conditions and received therapy as well as smoking and alcohol consumption. Post vaccination, potential AEs were recorded after each vaccine dose and anti-spike IgG responses were measured 3–4 weeks after the second dose (first period). To obtain an assessment of the durability of the antibody response, measurements were repeated three months after receipt of the second dose in 110 of 439 vaccine recipients (second period). Thus, this was a longitudinal (repeated measures) study.

The study was approved by the Medical Ethics Committee of the General Hospital of Arta. Written informed consent was obtained from study participants. All procedures contributing to this work comply with the ethical standards of the relevant national and institutional committees on human experimentation and with the Helsinki Declaration of 1975, as revised in 2008.

### Serological testing

Sera were stored at 2–8°C until testing for IgG antibodies against the RBD of the S1 region of SARS-CoV-2 using the Dimension^®^ EXL^™^ SARS-CoV-2 IgG (CV2G) assay (Siemens Healthineers), according to the manufacturer’s instructions. Results were expressed in index units and samples with IgG (anti-S-RBD)>1000 index were deemed positive. Sera from not infected or vaccinated individuals were used for the estimation of the cut-off. The sensitivity and specificity of the test are both reported by the manufacturer to be 100%.

### Statistical analysis

#### Software used in analysis

Data analyses were performed in R [16], except otherwise stated, by using or programming functions of the following libraries: “dplyr” [17], “tidyr” [18], “ggplot2” [19], “MKinfer” [20], “stats” [16], “lmr4” [21], “lmerTest” [22], “robustlmm” [23], “emmeans” [24], “performance” [25], “Simpsons” [26], “MuMIn” [27] and “simr” [28].

#### Dataset and exploratory analysis

We cleaned and curated the data using the “tidyr” and “dplyr” libraries in R. After cleaning, the dataset was constructed in the long format by gathering the two sampling periods (i.e., the “First” and “Second”) into one variable named “Sampling”, and spreading each symptom (i.e., baseline condition) variable into the corresponding dichotomous (Yes or No) variable. We also constructed three dichotomous variables, named “Smoking”, “Alcohol” and “Drugs”, with the latter referring to received medication at baseline for at least one condition unrelated to COVID-19. We explored the data for structure and outliers using the “ggplot2” library in R. For flexibility in analysis, we constructed an age categorical variable by classifying individuals in five decade-spanning groups as follows: “21–30”, “31–40”, “41–50”, “51–60”, and “61–72”.

#### Associations of antibody titers with demographic variables within each sampling period

Associations between antibody titers and age were examined separately for males and females in each sampling period by Spearman’s exact test (StatXact, Cytel, corp. Mass., USA) using a Monte Carlo sampling approach with a sample size of 10,000. Within each sampling period, we examined mean differences of variables of interest by t-tests, bootstrapping the corresponding pair variables 10,000 times, using the “boot.t.test” function of the “MKinfer” library in R. To prevent against inflation of the Type I error (rejecting the true null hypothesis purely by chance) as a result of multiple pair comparisons, we applied the Benjamini-Hochberg [29] correction using the p.adjust function with the “BH” argument of the “stats” library in R. To cross validate our results, we also applied the pairwise Mann–Whitney U-test with the same correction, using the “stats” library in R.

#### Modelling to assess factors affecting antibody titers between sampling periods

To assess the potential effects of different factors on antibody titers, we used a mixed effects linear model appropriate for our experimental design which involved repeated measures (dependent observations). In this model, Titer was the dependent variable and Age, Gender, Smoking, Alcohol and Drugs prescription were the explanatory variables. Random intercepts for the sampling interval and subjects were also included in the model to control for random effects of the two variables. The potential interaction between the Gender and Age variables was also examined. Sampling interval was the three-month span between the two sampling periods. Age was the subjects’ age in years. Gender was the subjects’ sex (Male or Female), whereas Smoking and Alcohol represented the subjects’ smoking and alcohol consumption behavior (Yes or No), respectively. Only the subset of individuals who were sampled twice was considered in the model (n = 110). The selection of the subset of individuals was based on the coverage of the full range of antibody titers (after the second vaccine dose), from low to intermediate to high. Mixed effect models were analyzed by the “lmer” function of the “lmerTest” library in R. The model residuals were tested for normality, homoscedasticity, independence and outlier effects. As the random effects were independent to each other, a diagonal covariance matrix was assumed for variance calculation. The mathematical justification for the addition of random effects in the model was examined by comparing the log likelihoods of the AIC values between models containing the random effect of interest and models lacking it, in a step by step process [30]. For model comparisons we used the AIC function of the stat library and for model significances we used the ANOVA function of the stat library. The model with the least AIC and a high significance was considered as the best fitting to the data. As data with multiple levels of random variation may suffer from hardly detectable contamination and outliers, we also used a robust method for modeling and parameter estimation, implemented in the “robustlmm” library in R. The potential effect of Simpson’s paradox on the Titer *vs*. AGE was examined for the Gender and Smoking variables by using the “Simpsons” library in R. Power analysis was performed by simulation using the powerSim() function of the simr R library, with 1000 bootstraps of a sample size of 220 and an α value of 0.05.

#### Associations of post-vaccination adverse events with demographic variables and antibody titers

To examine potential associations between local (pain, edema, erythema) or systemic AEs (fatigue, fever, headache, myalgias, arthralgias, lymphadenopathy) after vaccination with the “Gender”, “Smoking” or “Alcohol” variables, we used exact tests of independence in contingency tables in which the rows depicted the levels of the “Gender”, “Smoking” or “Alcohol” categories and the columns depicted the levels of the vaccine-AE variable of interest (e.g. regional pain). Exact tests were performed with the StatXact program of Cytel Studio (v. 9.0, Cambridge, Massachusetts, USA). The application of exact tests was necessary to overcome the problems of sparse or unbalanced contingency tables for which asymptotic methods are inappropriate [31].

## Results

### Characteristics of the study population

The demographic features of the study participants are described in Table 1.

**Table 1:** Demographic features of participants during the two study periods.

	First period*						Second period**					
	Males		Females		Total		Males		Females		Total	
Gender	n	%	n	%	n	%	n	%	n	%	n	%
Age group (years)												
**21–30**	15	42.9	20	57.1	35	100.0	5	50.0	5	50.0	10	100.0
		10.0		7.0		8.0		15.6		6.4		9.1
**31–40**	8	21.6	29	78.4	37	100.0	1	14.3	6	85.7	7	100.0
		5.3		10.0		8.4		3.1		7.7		6.4
**41–50**	46	27.7	120	72.3	166	100.0	9	19.1	38	80.9	47	100.0
		30.7		41.5		37.8		28.2		48.7		42.7
**51–60**	61	36.7	105	63.3	166	100.0	12	32.4	25	67.6	37	100.0
		40.7		36.3		37.8		37.5		32.1		33.6
**61–72**	20	57.1	15	42.9	35	100.0	5	55.6	4	44.4	9	100.0
		13.3		5.2		8.0		15.6		5.1		8.2
**Total**	150	34.2	289	65.8	439	100.0	32	29.1	78	70.9	110	100.0
		100.0		100.0		100.0		100.0		100.0		100.0

* 3–4 weeks after the second vaccine dose,

** ~3 months after the second vaccine dose.

In total, 439 subjects (65.8% females), with a mean age (±SD) of 48.6 (±9.51) (range: 24.0–72.0) years who were vaccinated with two doses of the BNT162b2 vaccine, were included in the first period of the study analysis. The vaccinees’ cohort comprised healthcare professionals (132 medical doctors, including a 72-year-old retired physician, as well as 180 nurses, 19 emergency medical service (EMS) staff, 13 laboratory technicians, four biochemists, three health visitors, and two pharmacists) and nonmedical personnel of the hospital (46 administrative/secretarial support personnel, 19 cleaning, sterilization and canteen staff, 16 technicians, and five information technology (IT) technicians). Of the 439 study participants, 34.6% (113 females/39 males, mostly middle-aged) had a history of underlying diseases, such as arterial hypertension and diabetes, and were receiving therapy (unrelated to COVID-19 and not including immunosuppressive medications) at baseline (S1A Fig). Most participants did not smoke or consume alcohol on a regular basis. Only ~16% of subjects (43 females/27 males), mainly middle-aged individuals, were smokers (S1B Fig). About half as many subjects (15 females/19 males, ~8%), distributed across all age groups, drank alcohol regularly (S1C Fig). Information on received therapy at baseline, smoking and drinking habits was missing for five study participants (4 females/1 male). In the second period of the study (three months after receipt of the second vaccine dose), antibody responses were measured in 110 of the 439 vaccine recipients (70.9% females), with a mean age (±SD) of 48.14 (±9.36) (range: 25–65) years.

### Vaccination safety data

No solicitated AEs were reported post vaccination by almost half (~49%) of study participants (n = 439). The mostly mild, local and systemic AEs that were observed within the first day after each vaccine dose are presented in S1 Table. Pain at the site of injection was the most commonly reported local reaction (24.8–40.5%), principally by middle-aged women (Table 2).

**Table 2:** Presentation of local and systemic adverse events (AEs) after the first and second dose of BNT162b2 vaccine and median (IQR) SARS-CoV-2 anti-S-RBD antibody titers 3–4 weeks after the second dose (first period) in the study population (n = 439).

		After 1^st^ dose	After 2^nd^ dose	
		n (%)	n (%)	Anti-S-RBD IgG
**Local AEs**				
	**Gender**			
	Male	47 (10.7)	25 (5.7)	66410 (36204)
	Female	133 (30.3)	92 (20.9)	57566 (37553)
	**Age group (years)**			
	21–30	13 (2.96)	7 (1.6)	55492 (14688)
	31–40	16 (3.64)	12 (2.7)	60889 (45476)
	41–50	78 (17.8)	46 (10.5)	49251 (42775)
	51–60	60 (13.7)	46 (10.5)	53681 (29425)
	>60	13 (2.96)	6 (1.4)	42206 (13090)
**Systemic AEs**				
	**Gender**			
	Male	19 (4.3)	30 (6.8)	54092 (31568)
	Female	52 (11.8)	130 (29.6)	66410 (32801)
	**Age group (years)**			
	21–30	10 (2.3)	9 (2.1)	39240 (28862)
	31–40	4 (0.9)	17 (3.9)	62765 (29114)
	41–50	32 (7.3)	70 (15.9)	56724 (30983)
	51–60	20 (4.6)	55 (12.5)	50449 (29968)
	>60	5 (1.1)	9 (2.1)	48121 (36052)

Systemic AEs were more pronounced following the 2^nd^ vaccine dose, again predominantly among middle-aged women; they most commonly included the following AEs after the 1^st^ and 2^nd^ dose, respectively: fatigue (4.1% and 9.6%), fever (1.8% and 9.3%), headache (5.5% and 7.7%), myalgias (1.1% and 7.5%)/arthralgias (0.9% and 4.6%), and chills (0.7% and 4.3%). No severe allergic reactions were observed.

### Adverse events associations with gender and baseline characteristics

We investigated likely associations between AEs and gender. After the first vaccine dose, 46 (30.7%) men and 133 (46.0%) women reported pain at the site of injection, while 104 (69.3%) men and 156 (54.0%) women did not report this AE. After the second vaccine dose, 23 (15.3%) men and 91 (31.5%) women reported regional pain, whereas 127 (84.7%) and 198 (68.5) men and women, respectively, did not. In both cases, a strong association was found between gender and regional pain by three exact tests of independence (Pearson’s chi-squared, likelihood-ratio, and Fisher’s exact test), indicating a significant trend for women to develop this reaction compared to men (p = 0.002 and p<0.001 for the first and second vaccine dose across tests, respectively).

We further examined the available baseline characteristics of study subjects (received therapy for underlying diseases, smoking and drinking habits) for possible associations with any AEs. Only received therapy at baseline showed statistically significant associations with the following reported AEs: weakness, dizziness, and regional pain. The association with weakness was observed after both vaccine doses (p = 0.004 and p = 0.005), whereas the association with dizziness, and regional pain was observed after the second vaccine dose only (p = 0.024 and p = 0.003, respectively, Pearson’s chi-squared test).

To control for the potential contribution of Gender and Age, we also examined the conditional associations of the above exploratory factors to the corresponding AEs. This analysis did not reveal an association with the “Weakness” variable after the first vaccine dose, a finding suggestive of gender or age effects. The association with “Regional pain” was also attenuated after the first vaccine dose (p = 0.049), suggesting a similar effect. In contrast, the “Weakness”, “Dizziness”, and “Regional pain” reactions had almost the same probability significances after the second vaccine dose (p = 0.005, p = 0.024, and p = 0.003, respectively, by Pearson’s chi-squared test), indicating a strong association between drug intake (prescription medication) at baseline and these AEs.

### Immunogenicity trends by gender and age

**Fig 1:**
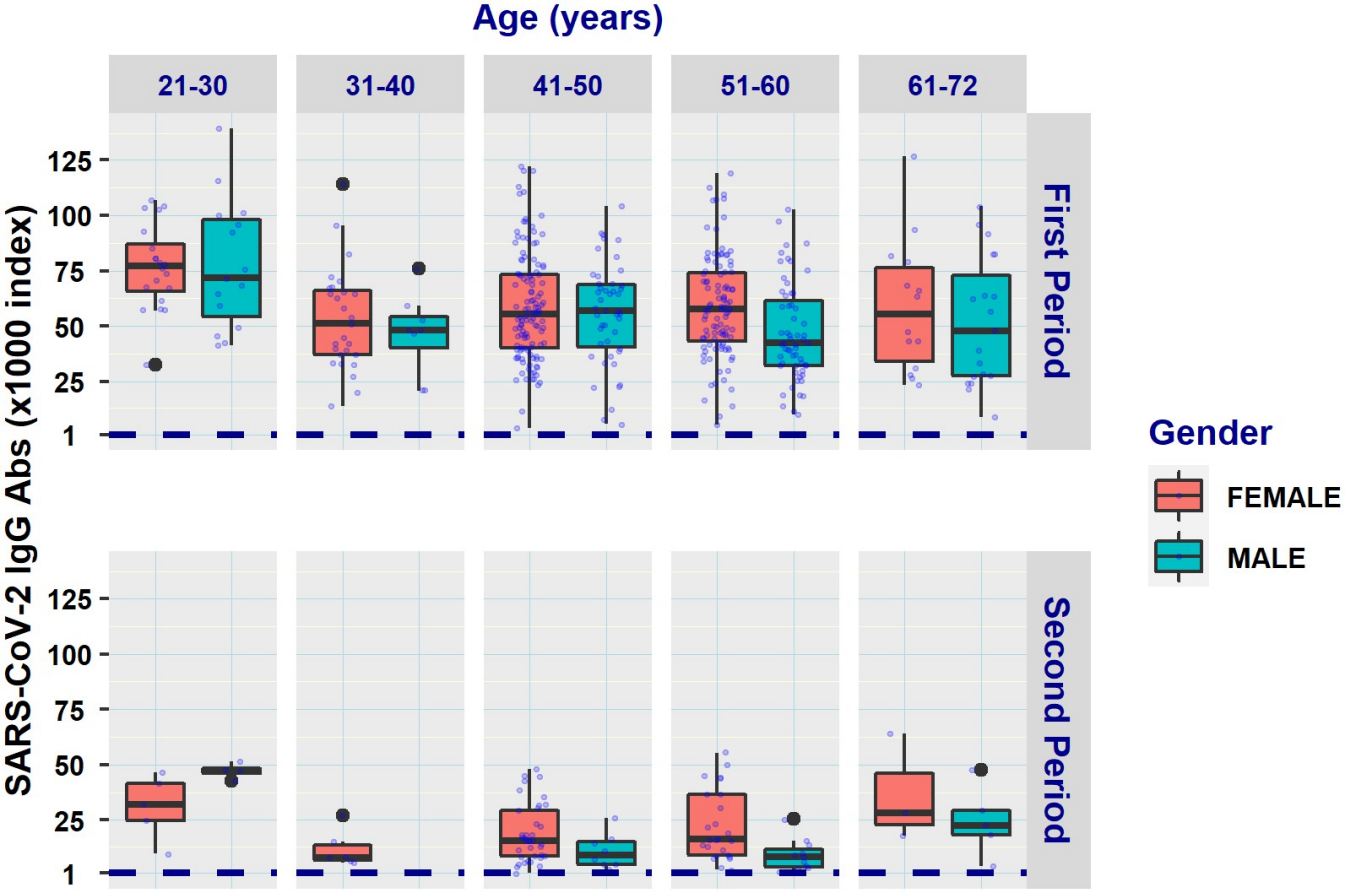
Box plots of SARS-CoV-2 anti-S-RBD antibody titers in females and males classified in different age groups by sampling period (First period: 3–4 weeks after the second vaccine dose; Second period: ~3 months after the second vaccine dose). Jittering was applied to dots for better visualization.

The results of the serological testing during the two study periods are shown in Table 3 and displayed graphically in Fig 1.

**Table 3:** SARS-CoV-2 anti-S-RBD IgG antibody titers by age and gender in the two study periods.

	1^st^ period (n = 439) *			2^nd^ period (n = 110) **		
Gender	Males (n = 150)	Females (n = 289)	Median by age	Males (n = 32)	Females (n = 78)	Median by age
Age group (years)						
21–30	71675 (43694)	76961 (21340)	75469 (33972)	47566 (4047)	32067 (16620)	44561 (13110)
31–40	48120 (13946)	53895 (30494)	51085 (29916)	18704 (0)	8140 (6834)	8557 (9908)
41–50	56743 (28351)	55258 (33491)	55790 (32954)	11135 (11535)	15707 (21638)	15670 (19419)
51–60	42494 (32395)	57637 (30542	53146 (33295)	8649 (10280)	16203 (27330)	13908 (17183)
61–72	44697 (40730)	63626 (43167)	47963 (52012)	22472 (11143)	46065 (41264)	28018 (29461)
**Median** **by gender**	50436 (33840)	58023 (35577)	56854 (34334)	15063 (26721)	16284 (23072)	16201 (23473)

* 3–4 weeks after the second vaccine dose.

** ~3 months after the second vaccine dose.

Median (IQR) values are shown.

Overall, lower titers were detected among participants in the second study period (~3 months after completion of the vaccination scheme) compared to the first (21,987 *vs*. 58,697).

Upon completion of the vaccination scheme (1^st^ period), female participants, who were also younger than male participants [mean (SD) age = 47.9 (8.9) *vs*. 49.9 (10.5) years, p = 0.0085], had higher antibody titers compared to males (60,967 *vs*. 54,325, p = 0.012). In the 2^nd^ period, females, who were only slightly younger than male participants [mean (SD) age = 47.8 (8.3) *vs*. 48.9 (11.7) years, p = 0.23], also had higher antibody titers compared to males, but these differences were not statistically significant (22,205 *vs*. 21,455, p = 0.50]. Mean antibody titers by gender and smoking for the two study periods are shown in Table 4.

**Table 4:** Mean anti-S-RBD IgG antibody titers by gender and smoking during the two study periods.

Period	Gender	Smoking	N	Mean anti-S-RBD IgG	SE
First	FEMALE	NO	67	70858	3746
First	FEMALE	YES	11	51110	7993
First	MALE	NO	28	54042	7184
First	MALE	YES	4	88948	4937
Second	FEMALE	NO	67	23222	2088
Second	FEMALE	YES	11	16013	3518
Second	MALE	NO	28	20693	3688
Second	MALE	YES	4	26788	7224

* 3–4 weeks after the second vaccine dose.

** ~3 months after the second vaccine dose.

A hint of interaction is apparent, as non-smoking females had higher titers than smoking females and non-smoking males had lower titers than smoking males (Table 4). Yet, no Simpson’s paradox effect was found on Titer vs. Age for either Gender or Smoking.

Regarding age, the youngest group (21–30 years) exhibited the highest antibody titers in both study periods, with mean values of 76,987 and 40,924 in the 1^st^ and 2^nd^ period, respectively. Lower titers were noted with increasing age for subjects >31 years, with a tendency for stabilization at a level of ~56,500 in the first period and, interestingly, a rebound to ~34,000 in the eldest (61–72 years) group in the 2^nd^ period.

### Contrasts analysis for gender and age differences

#### Gender differences among age groups

To further explore the impact of age on gender differences in the two periods, we examined contrasts (differences within each sampling period and gender category) by paired t-tests of all age category combinations, bootstrapping the paired variables 10,000 times. The results of this analysis are summarized in Table 5.

**Table 5:** Contrast pairwise comparison of mean anti-S-RBD IgG antibody titers (provided in S2 Table) of age groups by gender of study participants within each sampling period.

Age group (years)		Males		Females	
Age group 1	Age group 2	1^st^ period	2^nd^ period	1^st^ period	2^nd^ period
21–30	31–40	**0.0030**	NA*	**0.036**	0.083
21–30	41–50	**0.0086**	**p<0.001**	**0.0012**	0.23
21–30	51–60	**p<0.001**	**p<0.001**	**0.0034**	0.31
21–30	61–72	**0.012**	**0.013**	0.23	0.37
31–40	41–50	0.23	NA*	0.92	0.13
31–40	51–60	0.66	NA*	0.86	0.056
31–40	61–72	0.61	NA*	0.66	0.12
41–50	51–60	0.20	0.63	0.62	0.58
41–50	61–72	0.54	0.37	0.59	0.12
51–60	61–72	0.81	0.19	0.73	0.14

Probabilities by t-tests and bootstrapping are provided, with significant associations shown in bold.

*NA, Not Applicable, due to the limited sample size of the 31–40 age group category in the second period.

In the first period, the antibody titers of females declined with age up to 50 years from a maximum of 76,646 among the 21–30 years-old, and then increased again in the 51–60 and 61–72 age groups (S2 Table). Our contrasts analysis showed significant differences to exist among females between the 21–30 (youngest) and the 31–40, 41–50 and 51–60 age groups, but not between the 21–30 and the 61–72 group (76,646 *vs*. 63,466, Tables 3 and 4). In the second sampling period there were no statistically significant differences between the age groups of females. No other significant associations were found between the rest of the age categories of female participants in both periods.

In males, there were significant differences between the 21–30 group that displayed the highest titers (77,441 and 50,889 in the 1^st^ and 2^nd^ period, respectively) and the remaining age categories where antibody titers were lower in both sampling periods (Tables 3 and 5). No significant differences were found between any other combination of the age categories, although no estimation could be made for the 31–40 category that comprised of one individual only in the second period (Table 5).

The Mann–Whitney U-test showed the same significances with an exception in the comparison of the 21–30 versus the 31–40 age group which appeared non-significant in the first period for females (data not shown).

#### Age differences between genders

We also examined the impact of gender on differences among age groups within each sampling period by paired t-tests and bootstrapping (Table 6).

**Table 6:** Contrast pairwise comparison of mean anti-S-RBD IgG antibody titers (provided in S2 Table) of genders of study participants (males *vs*. females) by age within each sampling period.

Age group (years)	First period	Second period
21–30	0.91	**0.021**
31–40	0.15	NA*
41–50	0.45	0.56
51–60	**0.012**	0.12
61–72	0.25	0.20

Probabilities by t-tests and bootstrapping are provided, with significant associations shown in bold.

*NA, Not Applicable, due to the limited sample size of the 31–40 age group category in the second period.

In the first period, apart from the 21–30 age group where men surpassed slightly (mean difference = 795), women had higher titers across age groups, with a minimum and a maximum titer difference of 3,002 in the 41–50 and 12,603 in the 31–40 group, respectively (S2 Table). However, the only statistically significant titer difference (11,041) was observed in the 51–60 group (p = 0.012, Table 6).

In the second period, men had significantly higher titers in the 21–30 age group (p = 0.021) and a higher rate of titer decrease up to the age of 60 compared to women (S2 Table and Table 6). Thus, while males still had higher titers in the 31–40 and 41–50 age groups, females had higher antibody titers in the following two age groups. None of these differences were significant statistically. Interestingly, women in the 51–60 and 61–72 groups had higher titers than women in the 31–40 and 41–50 groups in both periods.

**Fig 2:**
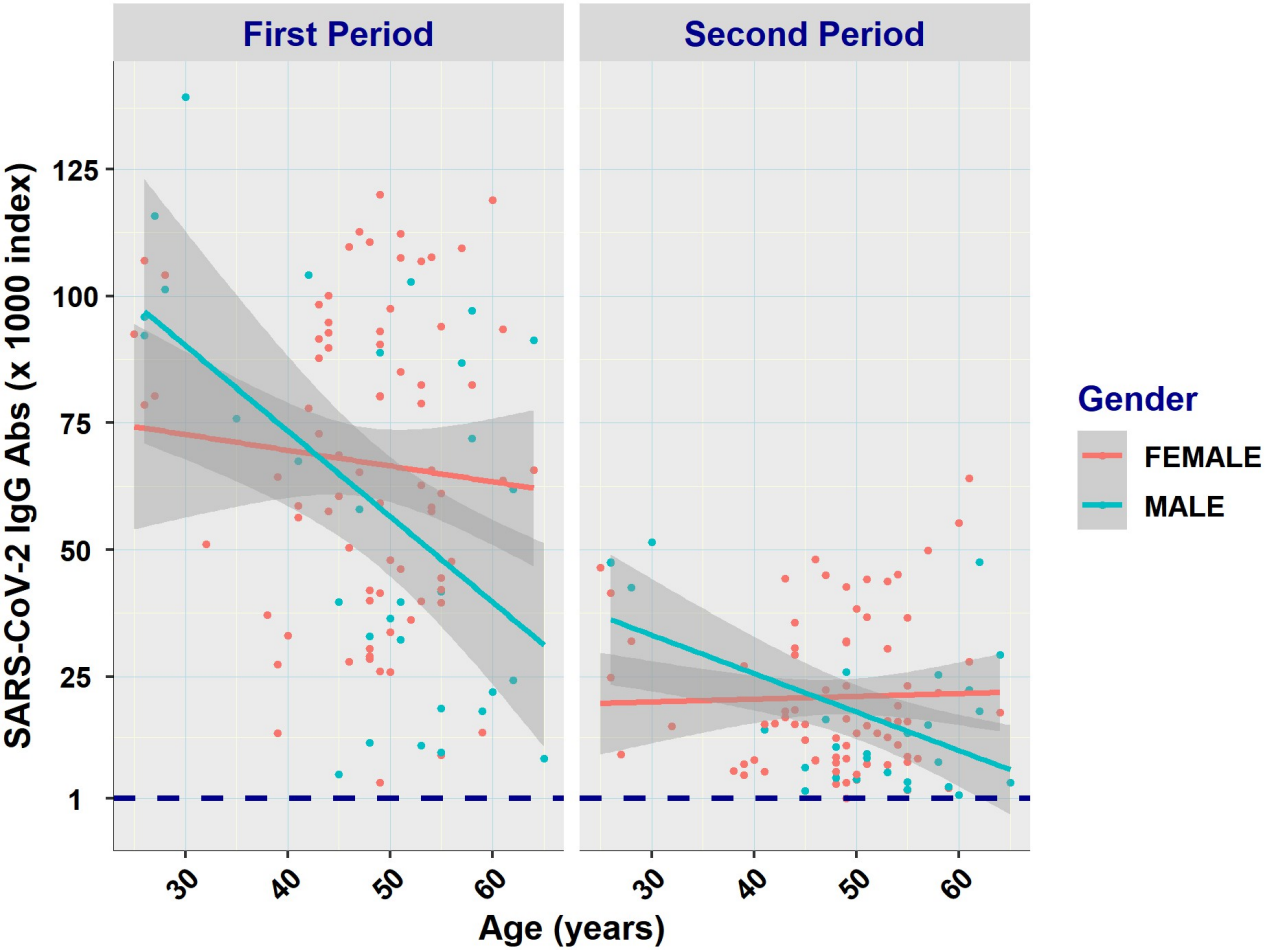
Least squares trend lines of SARS-CoV-2 antibody titers *vs*. age of females and males for each sampling period. The subset of subjects tested in both study periods (n = 110) was included here. Titers higher than three times the IQR were excluded as extreme outliers. Shaded areas represent 95% CIs. Approximately three months post vaccination, a trend of constant decline for males and a stabilizing process for females are evident. The coefficients of determination of each regression line were as follows: First period: Male R^2^ = 0.2767, Female R^2^ = 0.0071; Second period: Male R^2^ = 0.2894, Female R^2^ = 0.0001. In both periods females showed a consistent rate not differing from baseline.

These results are also consistent with the least squares trend lines of age *vs*. antibody titers created for each sampling period (Fig 2).

In the first period, although younger males had slightly higher titers than females, they also had a deeper negative slope and thus lower titers compared to females in ages >30 years. We used the non-parametric Spearman exact test to examine associations between variables. The null hypothesis (H0:ρ = 0) of the test indicates no correlation between the examined variables, while the alternative hypothesis (H1:ρ≠0) indicates a correlation between them. The corresponding correlations (Spearman exact test) between age and titer of males and females for the first period were -0.21 and– 0.07, respectively, and they were statistically significant for males (p = 0.011), but not for females (p = 0.27). In the second period, titers were reduced for both sexes. Intriguingly, however, females’ antibody titers were constant across ages, while males still had a negative slope of antibody titers. Males exhibited statistically significantly higher titers than females in younger and middle ages, but lower titers in ages >50 years. The corresponding correlations of male and female age groups with titers for the second period was -0.36 and- 0.07975, respectively, and they were marginally statistically significant (p = 0.043) and not significant (0.48), correspondingly.

### Model analysis of immune response associations with epidemiological and clinical features

Preliminary analysis by modelling using a mixed linear model included Titer as the dependent variable and demographic (Age, Gender) and health status indicators [Drugs (received therapy), Smoking, and Drinking] as the explanatory categorical variables. Alcohol consumption and received therapy at baseline had no significant effects on antibody titers (p>0.2) and they were thus removed from the model. A statistically significant interaction (p = 0.024) was found between Smoking and Gender. Hence, the selected model comprised of the antibody titer as the dependent variable, “Gender”, “Age” and “Smoking” as separate categorical explanatory variables with the Gender × Smoking interaction, and the individuals’ random effects for the two sampling periods. The reference in the model was the 21–30 age group of non-smoking females.

The assumptions of collinearity, influential observations (outliers), normality of residuals and normality of random effects were met by the data. Slight deviations were observed for the linearity and homogeneity of variance assumptions. The model without random effects (glm model) had had a higher AIC value compared to the same model considering random effects which had the lowest of all combinations, indicating the best fit of data. The statistical power of our study, for our sample size of 220 and alpha = 0.05, was found to be 98.30%, 95% CI (97.29, 99.01) using the simr package.

Mixed effects model analysis showed that there were highly significant differences (p<0.001) between the 21–30 years group (youngest vaccinees) and the 31–40, 41–50, and 51–60 groups, as well as a marginally significant difference (p = 0.092) between the 21–30 and the 61–72 years group (Table 7).

**Table 7:** Estimated IgG anti-S-RBD titers of the 110 subjects that were included in both study periods by the mixed effects model using the gender and age variables as predictors.

	Anti-S-RBD IgG	SEM	df	t	*P*
**Age group (years)**					
21–30 (Reference)	75,481	22813.86	1.24	3.31	0.15
31–40	-45,880	11291.71	102	-4.06	p<0.001
41–50	-30,653	7756.05	102	-4.00	p<0.001
51–60	-31,296	7828.25	102	-4.00	p<0.001
61–72	-17,121	10080.61	102	-1.70	0.092
**Gender**					
Male	-14,026	5058.32	102	-2.77	0.0066
**Smoking**					
Yes	-6,879	7411.99	102	-0.93	0.35
**Interaction**					
Smoking × Male	30,411	13716.71	102	2.22	0.024

SEM, standard error of the mean (explained variance); df, degrees of freedom; t, t-statistic, the ratio of departure of estimated from hypothesized values to standard error; *P*, probability of difference from zero.

**Fig 3:**
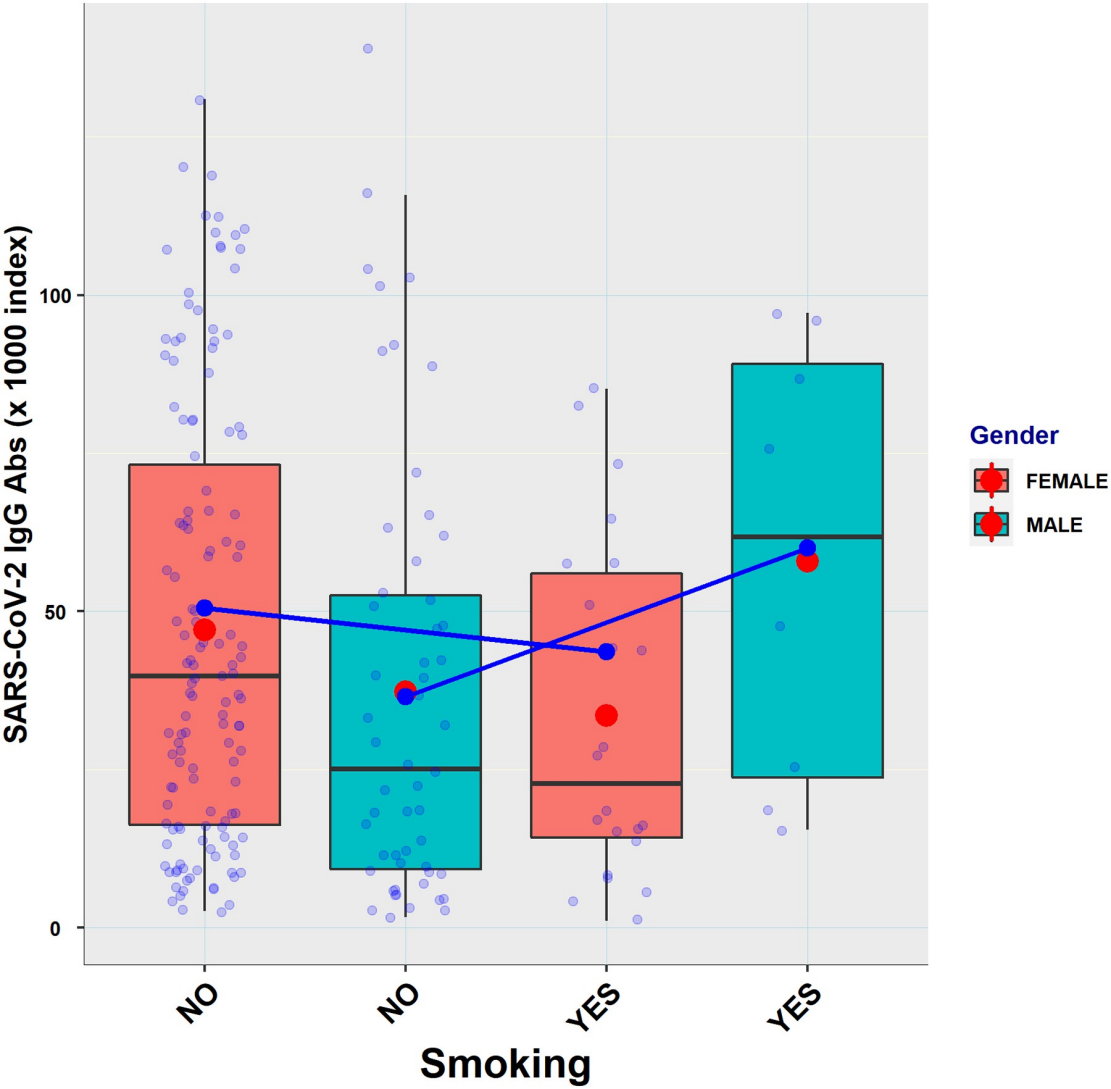
Boxplots of antibody titers of the two sampling periods (n = 220) shown by smoking and gender, and interaction lines between the two factors (p = 0.024). Horizontal black lines show the median; red dots show the sample means; filled blue dots show titer estimation determined by the model; open blue dots represent sample values. Random jittering was applied to help visualization of overlapping points.

In addition, the mixed effects model showed a higher antibody titer by 14,026 for females compared to males, which was statistically significant (p = 0.0066). However, since a weak interaction was found between Smoking and Gender (Table 7, Fig 3), more work is needed to shed light to the true differences between titers of males and females.

As determined by simple regression analysis using the same explanatory variables, the gender × smoking interaction was also significant in the first period in either the whole sample of study participants (p = 0.012, n = 439) or the subset of the sample used in the mixed effects model (p = 0.0091, n = 110). In the second period, although non-smoking females had higher titers than smoking females and non-smoking males had lower titers than smoking males (Table 4) the interaction was not significant (p = 0.26, n = 110).

Compared to the reference group, the 31–40 years age category had the lowest antibody titers with mean differences of 29,601 (75,481–45,880) for females and 15,575 (29,601–14,026) for males. The 41–50 and 51–60 age categories had almost equally reduced titers, with estimated values of about 44,828 (75,481–30,653) and 44,185 (75,481–31,296) for females, and 30,802 (44,828–14,026) and 30,159 (44,185–14,026) for males, respectively. The minimum difference was observed between the eldest (61–72 years) and the youngest (21–30 years) age category. Because of the slight violation of the homoscedasticity and linearity, we explored our model by using the “robustlmm” package. Slightly different estimates and almost the same probability values were obtained (data not shown). As the parameter estimation methods in this algorithm are robust as the name implies, the slight departure from the regression assumptions or other unobserved data contamination seems not to have had significant effects in our estimations. In support of our analysis, recent research showed no parameter bias in mixed effects models that violate the associated assumptions [32].

## Discussion

In this work, we scrutinized the associations of IgG antibody responses against the RBD of the spike protein of SARS-CoV-2 after immunization with two doses of the BNT162b2 vaccine in a cohort of HCWs (n = 439) and in a subset of vaccinees (n = 110) three months post-vaccination, with epidemiological, behavioral and clinical parameters. In particular, we explored possible associations of vaccination side effects after each vaccine dose to gender and investigated the potential role of exploratory variables on these effects. We also probed the potential associations between generated antibody titers of vaccinees approximately one and three months following the second vaccine dose, and the demographic variables of gender and age, and clinical and behavioral variables at baseline that included received medication (unrelated to COVID-19), alcohol consumption and smoking. Using a mixed effects model with titer as the dependent variable and age groups, gender, smoking and smoking-gender interaction as exploratory variables, we found differences in antibody titers between age groups as well as between genders and we also detected an interaction between smoking and gender.

The previously reported by many other studies [e.g. [13]], good general safety profile of the BNT162b2 vaccine was also confirmed in our cohort. Almost half of study participants reported no solicitated AEs post vaccination and no serious events were recorded. The most commonly reported local reaction was pain at the site of injection. We found a statistically significant association between female sex and this local AE, confirming previously reported observations. Systemic AEs were limited and more pronounced following the second vaccine dose, again chiefly among middle-aged women. This finding is not surprising given that profound differences between the sexes are known to exist to seasonal and pandemic influenza vaccines, for instance: antibody responses and vaccine efficacy, but also adverse reactions are higher in females compared to males [33].

We also found statistically significant associations between received therapy at baseline for underlying medical conditions (unrelated to COVID-19) and regional pain as well as dizziness and weakness. The associations with regional pain and dizziness held after the second vaccine dose only, whereas the systemic AE of weakness was evident after both vaccine doses. To the best of our knowledge, no detailed work has been done to study such associations. A more detailed approach is thus needed to exclude putative interacting or lurking variables to confirm this result. We cannot exclude the possibility of involvement of a particular drug or drug category, especially since in our study we examined prescribed medication as a pooled variable which included different types of drugs. A more detailed study is needed to possibly partition the observed association to different types of medication.

Humoral immune responses following vaccination with BNT162b2 against COVID-19 have been reported to be age-and gender-dependent, and more robust in younger ages and female octogenarians [34]. Nevertheless, not much work has been done on exploring the potentially differing antibody kinetics between genders. Our work corroborated previous findings of an overall advantage for younger people in immunogenicity and the negative effect of older age [10,11,13]. It also shows a clear predominance in antibody levels for females in most age categories, confirming previous observations [35,36]. As expected since antibody levels against the spike glycoprotein of SARS-CoV-2 wane over time [35,37], titers were higher for both genders one month after complete vaccination compared to the levels measured three months later. Despite the decline in anti-S and neutralizing antibody levels, memory B-cell populations may be maintained as observed after natural infection [38]. It is therefore unknown if this decrease also signifies the diminution of offered protection since a protective antibody threshold or the immunological correlates of protection for that matter, have not been defined yet [6].

One interesting observation is the disparity in the decreasing rate of titers with age between the two genders as evidenced by comparing the corresponding least squares trend lines for the two periods. Females exhibited a slower titer decrease rate across age one month after receipt of the second dose compared to males, ending up in lower, but almost constant levels three months after having the second dose. In contrast, males displayed a consistent decrease rate with age in both periods after vaccination, indicating a propensity of elderly males to have lower titers and vaccine responses compared to elderly females three months post vaccination. Although in most cases the differences between males and females were not statistically significant, these findings merit further investigation to understand sex-age differences in vaccine responses that could be important for guiding public health decisions.

Our analysis also showed an interaction between gender and smoking in antibody titers, such that women who smoked had lower antibody titers than women who did not, whereas men who smoked had higher antibody titers than nonsmoking men. Smoking, in general, is known to be associated with lower titers after vaccination against some viruses such as *Influenza A virus* [39]. However, an increase of antibody titers in smokers compared to nonsmokers has also been reported for some strains of *Influenza A virus* [40,41]. Recent studies on COVID-19 vaccines report a negative effect of smoking on antibody titers after BNT162b2 vaccination [13]. In our observations, although an effect of smoking on antibody titers cannot be ruled out, the involvement of additional, uncontrolled lurking variables, should be considered before inferring cause-effect explanations of the observed association of smoking to antibody levels. Indeed, uncontrolled variables associated with smoking may create a relationship to the response variable (titer) through the gender (male and/or female) association to the response, which could be mistaken as a real effect. A more thorough experimental approach is thus needed to shed light to the nature of the observed interaction.

Limitations of our study include the unavailability of serological testing of all study participants in the second period, the overrepresentation of females and the imbalanced distribution of subjects across age groups, as well as the lack of assessment of additional antibody classes (IgM, IgA) or other components of immune responses, such as neutralizing antibodies and cell-mediated immunity. However, issues that could have distorted our results have been avoided through our meticulous analysis of available data. The next rounds of results of this prospective study, six- and nine months post vaccination, will shed additional light to the value of identified associations of antibody titers with epidemiological, behavioral and clinical parameters.

## Supporting information

S1 Fig Health status indicators of study participants by age and sex at baseline (5 missing values, 4 females/1 male). **A.** Received therapy. **B.** Smoking. **C.** Alcohol consumption. Of the 439 participants, 287 had no underlying disease, while the distribution of conditions among the remaining 152 subjects (25 of whom reported more than one conditions) was as follows: Arterial hypertension 41, thyroid abnormalities 33, diabetes 25, cardiovascular diseases 22, autoimmune diseases 19, asthma 11, cancer 5, arthritis 5 and other diseases (e.g. glaucoma, urticaria) 16.
(PDF)

S1 Table Local and systemic adverse events (AEs) from 439 HCWs after the 1^st^ and 2^nd^ dose of BNT162b2 vaccine. (DOCX)

S2 Table SARS-CoV-2 anti-S-RBD IgG antibody titers by age and gender in the two study periods. Mean (SD) values are shown.
(DOCX)

S1 File Data set underlying the results described in this manuscript. (XLSX)
