# Supplementary material for: Age and sex associations of SARS-CoV-2 antibody responses post BNT162b2 vaccination in healthcare workers: A mixed effects model across two vaccination periods
Source: PLoS One. 2022 Apr 29;17(4):e0266958. doi: 10.1371/journal.pone.0266958 (PMC9053797; doi:10.1371/journal.pone.0266958)
Supplement: S1 Fig — A. Received therapy. B. Smoking. C. Alcohol consumption. Of the 439 participants, 287 had no underlying disease, while the distribution of conditions among the remaining 152 subjects (25 of whom reported more than one conditions) was as follows: Arterial hypertension 41, thyroid abnormalities 33, diabetes 25, cardiovascular diseases 22, autoimmune diseases 19, asthma 11, cancer 5, arthritis 5 and other diseases (e.g. glaucoma, urticaria) 16. (PDF) [file pone.0266958.s001.pdf]

SI Figure 1. Health status indicators of study participants by age and sex at baseline (5 missing values, 4 females/1 male). **A.** Received therapy. **B.** Smoking. **C.** Alcohol consumption.

A.

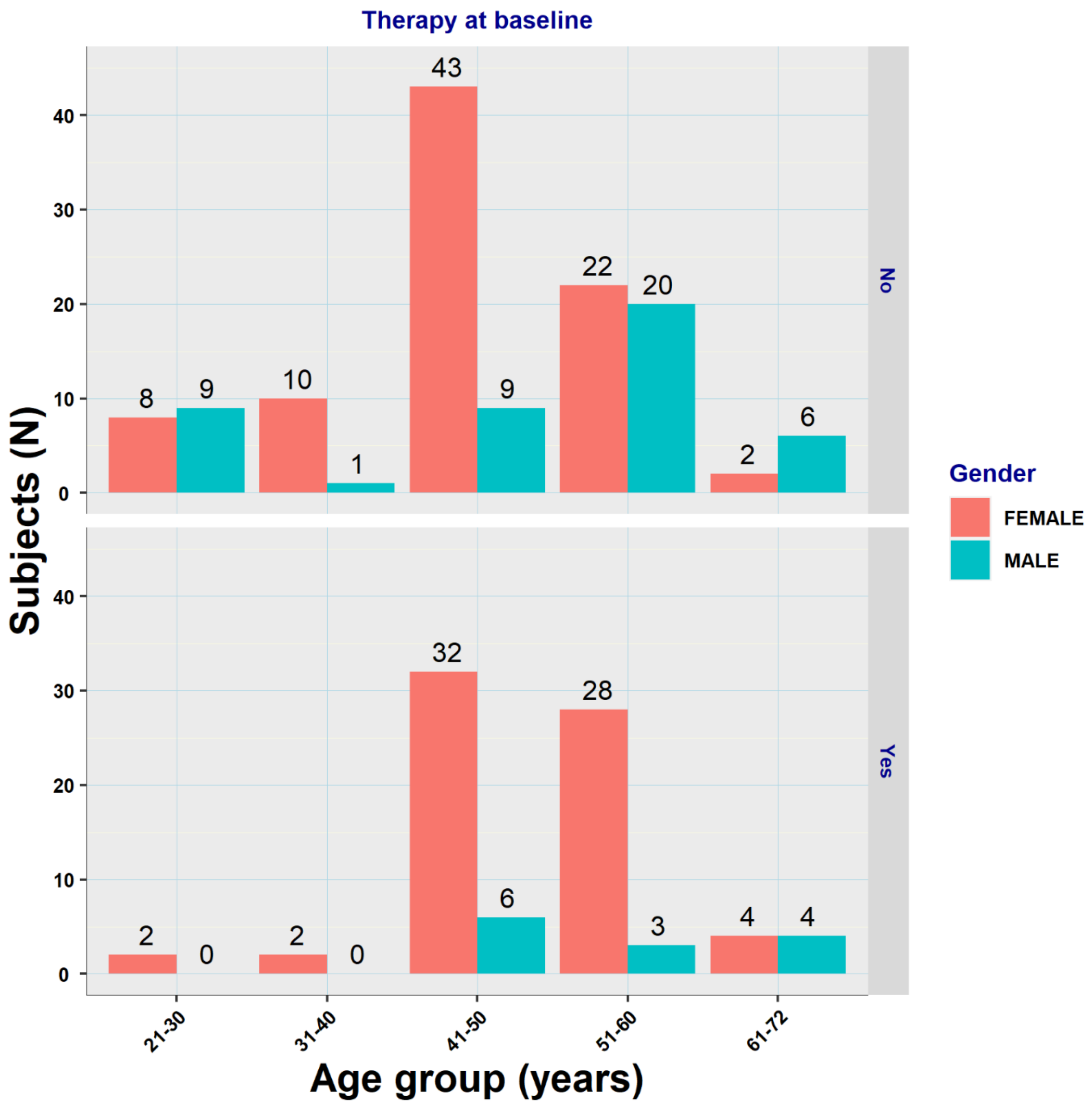

B.

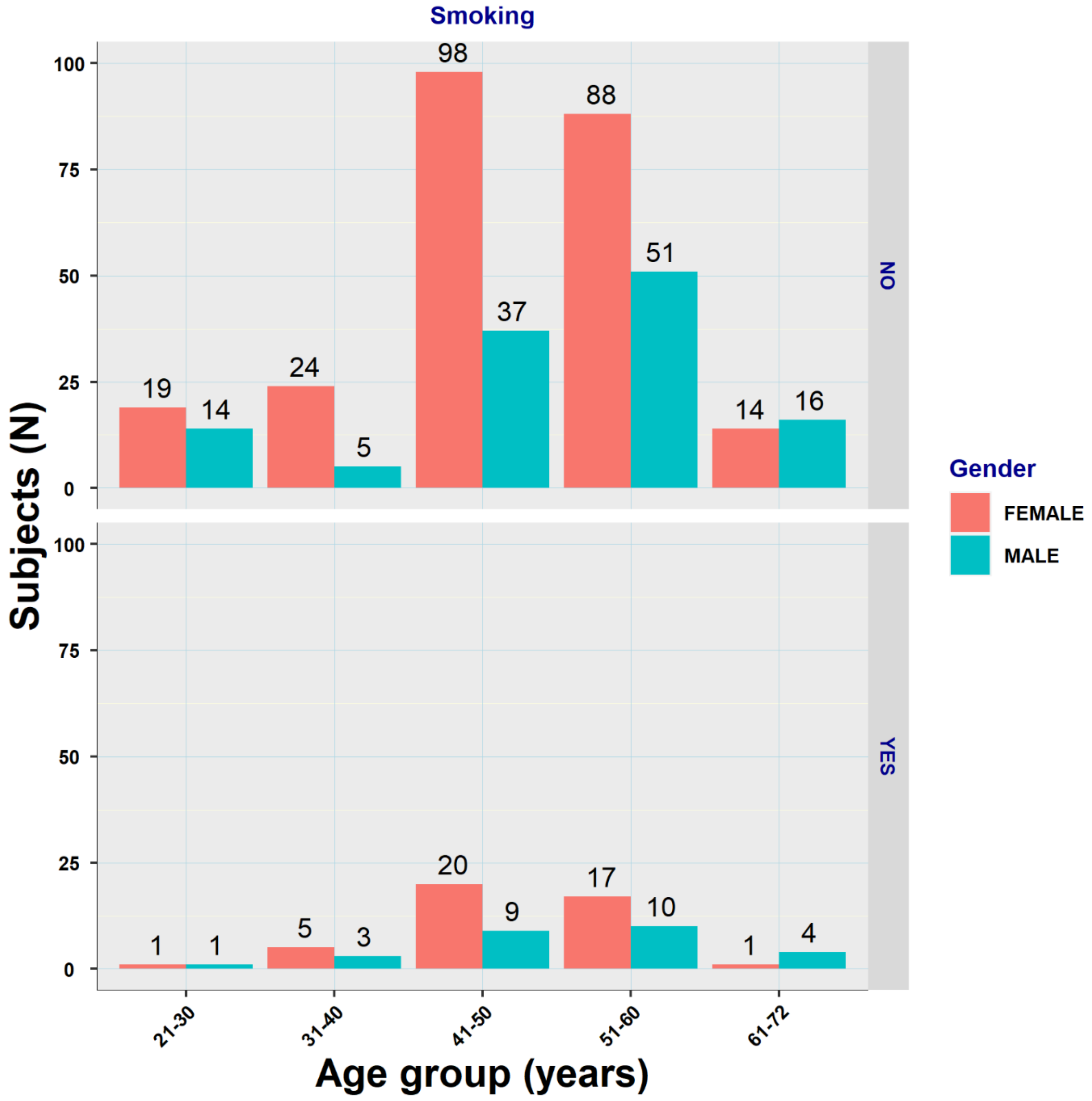

Smoking
100
75
50
25
0
98
88
51
37
19
14
24
5
14
16
NO
100
75
50
25
0
20
17
10
9
1
1
5
3
1
4
YES
Subjects (N)
21-30
31-40
41-50
51-60
61-72
Age group (years)
Gender
FEMALE
MALE

C.

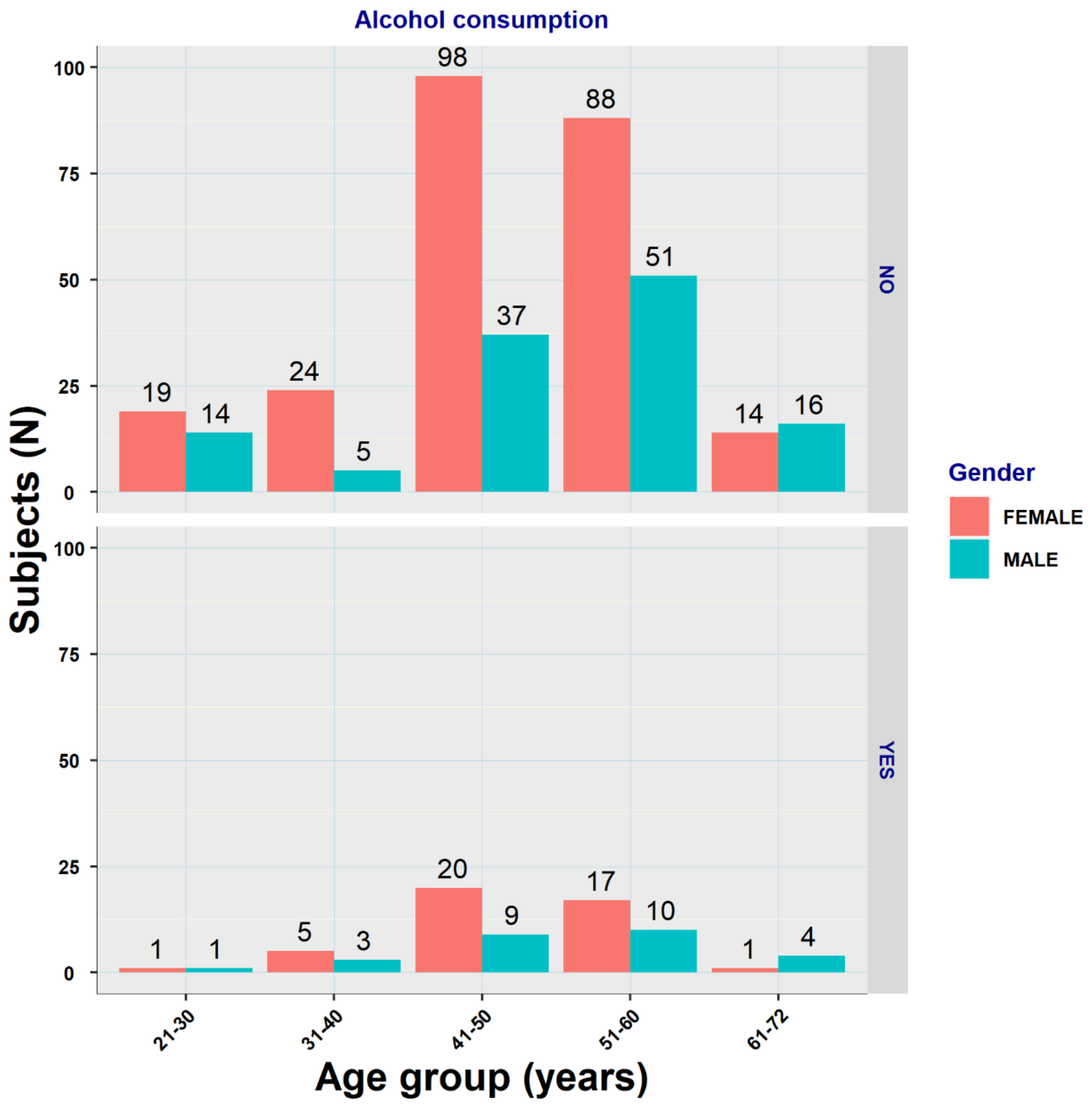
Alcohol consumption
Subjects (N)
100
75
50
25
0
19
14
24
5
98
37
88
51
14
16
NO
1
1
5
3
20
9
17
10
1
4
YES
21-30
31-40
41-50
51-60
61-72
Age group (years)
Gender
FEMALE
MALE
