# Supplementary material for: Age and sex associations of SARS-CoV-2 antibody responses post BNT162b2 vaccination in healthcare workers: A mixed effects model across two vaccination periods
Source: PLoS One. 2022 Apr 29;17(4):e0266958. doi: 10.1371/journal.pone.0266958 (PMC9053797; doi:10.1371/journal.pone.0266958)
Supplement: S1 Table — (DOCX) [file pone.0266958.s002.docx]

**S1 Table.** Local and systemic adverse events (AEs) from 439 HCWs after the 1^st^ and 2^nd^ dose

of BNT162b2 vaccine.

| **Adverse events** | **After 1^st^ dose**  **n (%)** | **After 2^nd^ dose**  **n (%)** |
| --- | --- | --- |
| **Local** | | |
| Regional pain | 178 (40.5) | 109 (24.8) |
| Edema | 2 (0.5) | 8 (1.8) |
| **Systemic** | | |
| Fatigue | 18 (4.1) | 42 (9.6) |
| Headache | 24 (5.5) | 34 (7.7) |
| Myalgias | 5 (1.1) | 33 (7.5) |
| Arthralgias | 4 (0.9) | 20 (4.6) |
| Fever | 8 (1.8) | 41 (9.3) |
| Chills | 3 (0.7) | 19 (4.3) |
| Nausea | 1 (0.2) | 3 (0.7) |
| Diarrhea | 2 (0.5) | 3 (0.7) |
| Vomiting | 2 (0.5) | 2 (0.5) |
| Dizziness | 10 (2.3) | 8 (1.8) |
| Drowsiness | 8 (1.8) | 5 (1.1) |
| Swollen lymph nodes | 1 (0.2) | 5 (1.1) |
| Wheeziness | 1 (0.2) | 1 (0.2) |
