# Supplementary material for: Age and sex associations of SARS-CoV-2 antibody responses post BNT162b2 vaccination in healthcare workers: A mixed effects model across two vaccination periods
Source: PLoS One. 2022 Apr 29;17(4):e0266958. doi: 10.1371/journal.pone.0266958 (PMC9053797; doi:10.1371/journal.pone.0266958)
Supplement: S2 Table — Mean (SD) values are shown. (DOCX) [file pone.0266958.s003.docx]

**S2 Table.** SARS-CoV-2 anti-S-RBD IgG antibody titers by age and gender in the two study periods. Mean (SD) values are shown.

|  | **First period (n=439) *** | | | **Second period (n=110) **** | | |
| --- | --- | --- | --- | --- | --- | --- |
| **Gender**  **Age group**  **(years)** | **Males**  **(n=150)** | **Females (n=289)** | **Mean**  **by age** | **Males**  **(n=32)** | **Females**  **(n=78)** | **Mean**  **by age** |
| 21-30 | 77,441  (29,179) | 76,646 (19,107) | 76,987 (23,554) | 50,889  (8,683) | 30,959 (14,517) | 40,924 (15,411) |
| 31-40 | 46,591 (18,361) | 59,194 (32,026) | 56,469 (29,849) | 18,704 (NA) | 11,774 (8,247) | 12,764 (7,971) |
| 41-50 | 55,811 (22,971) | 58,813  (24,572) | 57,981 (24,108) | 15,637  (15,768) | 20,126 (14,900) | 19,266 (15,000) |
| 51-60 | 49,534  (25,449) | 60,575 (2,562) | 56,517 (26,034) | 12,585  (13,821) | 22,279 (15,766) | 19,135 (15,661) |
| 61-72 | 51,277 (28,193) | 63,466 (34,237) | 56,501 (31,053) | 24,331 (15,943) | 46,196 (27,467) | 34,049 (23,298) |
| **Mean**  **by gender** | 54,325  (26,186) | 60,967  (26,211) | 58,697  (26,362) | 21,455  (18,870) | 22,205  (16,565) | 21,987  (17,182) |

* 3-4 weeks after the second vaccine dose

** ~3 months after the second vaccine dose
